# Functional Characterization of a Missing Branch Component in *Haematococcus pluvialis* for Control of Algal Carotenoid Biosynthesis

**DOI:** 10.3389/fpls.2017.01341

**Published:** 2017-08-02

**Authors:** Yong M. Lao, Hui Jin, Jin Zhou, Huai J. Zhang, Zhong H. Cai

**Affiliations:** ^1^Shenzhen Public Platform of Screening and Application of Marine Microbial Resources Guangdong, China; ^2^The Division of Ocean Science and Technology, Graduate School at Shenzhen, Tsinghua University Shenzhen, China; ^3^School of Life Sciences, Tsinghua University Beijing, China

**Keywords:** *Haematococcus pluvialis*, lycopene cyclase, branch point, carotenoid biosynthesis, environmental stresses, metabolic flux

## Abstract

Cyclization of acyclic lycopene by cyclases marks an important regulatory point in carotenoid biosynthesis. Though some algal lycopene epsilon cyclases (LCYEs) have been predicted computationally, very few have been functionally identified. Little is known about the regulation mechanisms of algal LCYEs. Recent comparative genomic analysis suggested that *Haematococcus pluvialis* contained only the β type cyclase (HpLCYB). However, in this study, carotenoid profiling found trace α-carotene in the salt-treated cells, indicating the *in vivo* activity of HpLCYE, a missing component for α-branch carotenoids. Thus, genes coding for HpLCYB and HpLCYE were isolated and functionally complemented in *Escherichia coli*. Substrate specificity assays revealed an exclusive cyclization order of HpLCYE to HpLCYB for the biosynthesis of heterocyclic carotenoids. Expression pattern studies and bioinformatic analysis of promoter regions showed that both cyclases were differentially regulated by the regulatory *cis*-acting elements in promoters to correlate with primary and secondary carotenoid biosynthesis under environmental stresses. Characterization of the branch components in algal carotenoid biosynthesis revealed a mechanism for control of metabolic flux into α- and β-branch by the competition and cooperation between HpLCYE and HpLCYB; and supplied a promising route for molecular breeding of cyclic carotenoid biosynthesis.

## Introduction

**FIGURE 1:**
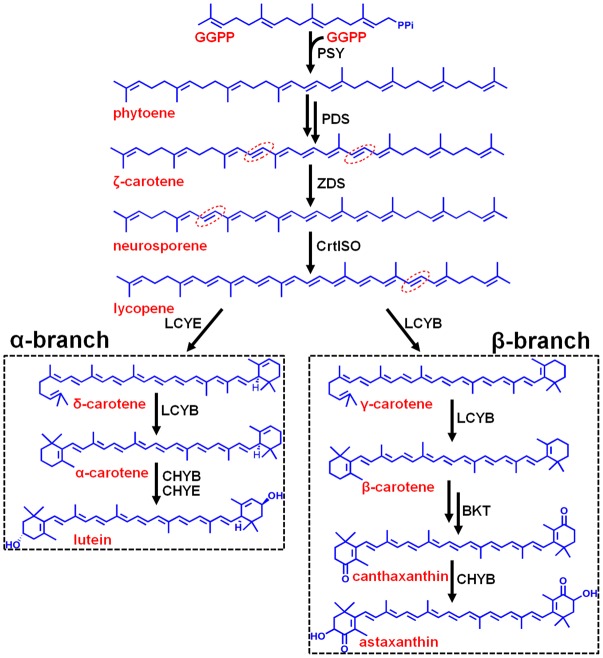
Hypothetic carotenoid biosynthesis pathway in *Haematococcus pluvialis*. Note that the branch point component HpLCYE was missing before this study, which controls α-branch composition in carotenoid biosynthesis. The 7–8, 7′–8′, 11–12, and 11′–12′ carbon-carbon double bonds are shown by red dotted line cycles. Enzymes synthesizing the corresponding carotenoid intermediates were given at the side of the arrows. GGPPS, GGPP synthase; PSY, phytoene synthase; PDS, phytoene desaturase; ZDS, ζ-carotene desaturase; CrtISO, carotene isomerase; LCYB, lycopene β cyclase; LCYE, lycopene ε cyclase; BKT, β-carotene ketolase; CHYB, β-carotene hydroxylase; CHYE, ε-carotene hydroxylase; GGPP, geranylgeranyl diphosphate.

Carotenoids are essential components in all photosynthetic apparatus of plants, algae, and cyanobacteria, fulfilling crucial functions such as photoprotection as antioxidant by quenching of triplet chlorophylls and scavenging of various reactive oxygen species (ROS), and photosynthesis as accessory light-harvesting pigments in the photosynthetic antenna complexes (Lao et al., 2011). Natural carotenoids can be chemically divided into carotenes and xanthophylls according to whether they contain oxygen. Carotenes are a kind of hydrocarbons that include α-, β-carotene, and lycopene (Christaki et al., 2013). Xanthophylls are oxygenated molecules with oxygen being present as hydroxyl groups (e.g., lutein) or as oxo-groups (e.g., canthanxanthin) or as a combination of both groups (e.g., astaxanthin) (Jackson et al., 2008). Carotenoid biosynthesis begins with sequential synthesis of a series of acyclic carotenes, e.g., phytoene, ζ-carotene, neurosporene, and lycopene, from the condensation of two geranylgeranyl diphosphate (GGPP) molecules (**Figure 1**). These steps constitute the most common part of carotenoid biosynthesis in photosynthetic organisms.

Most carotenoids have a cyclic structure at one or both end(s), commonly found in plants and algae are β- and ε-ring, and their derivatives thereof. These two types of cyclic end groups only differ in the position of the double bond within the cyclohexene ring. Adding a β- or ε-ionone ring to one or both ends of lycopene results in the formation of γ- and β-carotene, or δ- and ε-carotene, respectively; while consecutively adding a β- and a ε-ionone rings to either end of lycopene forms heterocyclic α-carotene (**Figure 1**). Carotenoids with two β-rings, e.g., β-carotene, are ubiquitous and serve primarily in protecting against photooxidation and/or in dissipation of excess light energy (Cunningham and Gantt, 2001). Carotenoids with one β- and one ε-ring, e.g., lutein, are the predominant carotenoid in the light-harvesting antenna of plants and algae (Cunningham and Gantt, 2001). Carotenoids containing two ε-rings are not common, with the exception of lettuce and maize which produce a substantial amount of ε,ε-carotene (ε-carotene) (Phillip and Young, 1995; Bai et al., 2009).

Cyclization of acyclic lycopene is a vital branch point in carotenoid biosynthesis. It directs substrate towards the synthesis of β-branch products by lycopene β-cyclase (LCYB) and α-branch products by lycopene ε-cyclase (LCYE), respectively (**Figure 1**). LCYB and LCYE are closely related membrane-associated enzymes. Most LCYBs are bicyclase that consecutively introduce a β-ring to both ends of lycopene to form bicyclic β-carotene as a final product through monocyclic γ-carotene; and to introduce a β-ring to the linear end of monocyclic δ-carotene to form heterocyclic α-carotene. Whereas, most LCYEs do not accept cyclic carotenoids like δ- and γ-carotene as substrates, they are monocyclase that introduce a single ε-ring to either end of lycopene to form δ-carotene. However, exceptions were found recently in *Myxococcus xanthus* (Teramoto et al., 2003) and *Rhodococcus erythropolis* (Tao et al., 2004) which contain LCYBs with monocyclase activity that introduce only one β-ring to lycopene, and in *Lactuca sativa* (Phillip and Young, 1995) and *Zea mays* (Bai et al., 2009) which contain bicyclase LCYEs that adds two ε-rings to both ends of lycopene.

Currently, most of the algal LCYE sequences are obtained by sequence alignment. Only a few have been functionally identified as authentic LCYE enzymes, including *Chlorella* (*Chromochloris*) *zofingiensis* LCYE (Cordero et al., 2012) and *Ostreococcus lucimarinus* LCYE (Blatt et al., 2015). Little is known about the regulation mechanisms of algal LCYEs. The green alga *Haematococcus pluvialis* surpasses any other reported sources to massively accumulate large amounts of astaxanthin in response to a variety of environmental stresses, such as high light, nutrient starvation, high salinity and oxidative stress, etc (Boussiba, 2000). Extensive works on carotenoid profiling and key genes in carotenogenesis have been carried out in *H. pluvialis* (Kobayashi et al., 1993; Tripathi et al., 1999; Steinbrenner and Linden, 2001, 2003; Denery et al., 2004). Recently, a comparative genomic analysis of algal lycopene cyclases suggested that *H. pluvialis* only contained the β type cyclase due to gene lost in evolution (Cui et al., 2011).

However, by carotenoid profiling in the *H. pluvialis* cells imposed by environmental stresses, we found here a trace amount of α-carotene in NaCl-treated cells, indicating a missing LCYE responsible for α-branch products. The gene encoding HpLCYE was isolated and functionally identified. Regulation of HpLCYE and HpLCYB were investigated at the transcriptional, protein, and metabolic levels. Expression pattern studies, bioinformatic analysis of promoter regions, and substrate specificity assays suggested a mechanism for control of cyclic carotenoid composition by the complexity of regulation of the two cyclases.

## Materials and Methods

### Strains and Cultivation Conditions

Algal cells were normally incubated in conical flasks containing 100 mL of BG11 medium in an illumination incubator at 25°C for constant illumination (1,000 lx of cool white fluorescent lights) under a 12 h light :12 h dark cycle, and were shaken manually twice daily. Cells grown to the logarithmic phase were collected by centrifugation at 8,000 *g* for 15 min at 25°C. Then, ∼10^9^ cells were transferred to fresh BG11 medium, and were cultured under stress conditions at 25 °C for 30 days. Specifically, for dark stress, cells were cultured in a black chamber; for high light stress, up to 6,000 lx cool white fluorescent light was imposed; for salt stress, BG11 containing 42 mM NaCl was used; for oxidative stress, BG11 containing 450 μM FeSO_4_ and 45 mM sodium acetate (SA) was used; for nutrient starvation, algal cells were washed three times using nitrogen-free (N^-^) or phosphorus-free (P^-^) BG11, and inoculated into fresh N^-^ and P^-^ BG11, respectively. Cells cultured under normal conditions were used as control. Also, cells were shaken manually twice daily.

Carotenoid-accumulating *E. coli* DH5α strains were cultured in LB medium containing 100 μg mL^-1^ ampicillin at 30°C in dark with shaking at the speed of 220 rpm for 48 h. Then, cells were transferred to a back chamber to completely induce carotenoid synthesis at 25°C for another 48 h.

### Pigments Extraction and Saponification

Algal cells equivalent to 100 mg dry weight were collected by centrifugation at 8,000 *g* for 10 min at 25°C, lyophilized, and were weighed, sequentially. The dry cells were resuspended in 10 mL of 1 M cool chloroform, and were sonicated at 4°C for 20 min. The broken cells were vigorously mixed, incubated on ice for 30 min, and then were centrifuged at 12,000 *g* for 15 min at 4°C. The upper layer was collected. Second extraction was carried out and the supernatants were then merged, evaporated to dryness, and were dissolved in 5 mL of acetonitrile. The extracts were stored at –80°C until use. Then, 100 μL of extracts were diluted with 2 mL of acetonitrile, and were hydrolyzed by adding 10 μL of 1 M NaOH. Saponification was proceeded for 6 h at 4°C in dark. The saponified extracts were then washed several times with distilled water until the pH was neutral, and were analyzed by UPLC directly.

∼150 mL of *E. coli* DH5α cells were harvested by centrifugation at 12,000 *g* for 5 min at 4°C, and were incubated at a water bath at 55°C for 15 min with vigorous shaking at a 5 min-interval after addition of 3 mL of acetone. Then the supernatants were collected by centrifugation at 12,000 *g* for 15 min at 4°C, and were subsequently evaporate to dryness, and were dissolved in 2 mL of acetonitrile for UPLC assay.

### UPLC Analysis

A Waters ACQUITY UPLC^TM^ H-CLASS equipped with a quaternary pump, an autosampler, a column oven and a PDA detector was used for carotenoid profiling. Carotenoids were separated at the flow rate of 0.4 mL min^-1^ on a Waters BEH C18 column (2.1 mm × 50 mm, 1.7 μm) using methanol (A) and acetonitrile (B). The gradient elution was 10% A, 90% B at 0 min, followed by a linear gradient to 0% A and 100% B to 4 min, maintained at 0% A and 100% B to 12 min, returned to the initial condition by 12.1 min, re-equilibrated at the initial condition by 15 min. The injection volume was 5 μL. Needle was washed using acetonitrile/methonal (9 : 1; V : V) mixture for 10 s after each injection. Column temperature was maintained at 35°C using a column oven. The detection of analysts was carried out by ultraviolet (UV) absorbance at 450 nm. The filter constant was set to 0.2. All system controls and data analyses were processed by the Empower 3 software.

### Extraction of Genomic DNA and Total RNA

Genomic DNA extraction from algal cells in the logarithmic phase was performed using the E.Z.N.A.^TM^ High Performance (HP) Plant DNA Kit (OMEGA, China) according to the manufacturer’s recommendation. Total RNA was extracted from ∼10^7^ *H. pluvialis* cells grown at the logarithmic phase using the TRIzol^®^ reagent (Life Technologies, United States) following the conditions recommended by the manufacturer.

### Isolation of the *H. pluvialis* Cyclase cDNAs

Degenerate primers for the isolation of expressed sequence tag (EST) were designed according to two pairs of conserved regions retrieved from sequence alignment of known plant and algae cyclases, i.e., upstream “PTFLYAMP” and downstream “AGMVHPSTG” for *HpLcyB*, and upstream “NNYGVW” and downstream “SYIPVGGPLP” for *HpLcyE*. Revers transcription-polymerase chain reaction (RT-PCR) was performed by the RNA PCR Kit (AMV) Ver.3.0 (TaKaRa, China). All manipulations followed to the manufacturer’s protocol. The parameters of PCRs were set as follows: 95°C, 5 min; 30 cycles of 95°C, 30 s, 48°C, 30 s, and 72°C, 1 min with a final extension at 72°C for 10 min.

Based on the EST fragments, two gene specific primers (GSPs) were designed and 3′ rapid amplification of cDNA ends (RACE) reactions were conducted using the designed GPSs and oligo dT-Adaptor primer. The parameters of PCRs were set as follows: 95°C, 5 min; 30 cycles of 95°C, 30 s, 50°C, 30 s, and 72°C, 1 min with a final extension at 72°C for 8 min.

5′ RACE reactions were accomplished using the SMARTer^®^ RACE 5′/3′ Kit (Clontech, United States) and two pairs of GSPs designed based on the obtained partial sequences. All manipulations were according to the user manual.

The full-length cDNAs were finally isolated using GPSs complementary with the 5′ and 3′ ends of the two cyclases gene, respectively. The parameters of PCRs were set as follows: 95°C, 5 min; 30 cycles of 95°C, 30 s, 55°C, 30 s, and 72°C, 2 min 30 s with a final extension at 72°C for 10 min. The amplified sequences were cloned into the *Bam*HI and *Hind*III sites of pET-32a-c(+) vector (Merck, German) and sequenced. The primers used in this article are listed in Supplementary Table S1.

### Promoter Isolation

To isolate the promoters of *HpLcyB* and *HpLcyE*, genome walking was carried out using the Universal GenomeWalker^TM^ 2.0 Kit (Clontech, United States). GSPs were designed according to the 5′ ends of the cDNA sequences under the instructions of the kit. All manipulations were according to the manufacturer’s recommendation.

### Molecular Evolution and Sequence Analysis

Sequence analysis was performed using BLAST Software^1^. Multiple alignments were conducted using Clustal X 1.83. Molecular evolutionary analysis were conducted using the Neighbor-Joining (Saitou and Nei, 1987) and Maximun-Likelihood methods (Jones et al., 1992), respectively, by the molecular evolution genetics analysis (MEGA) software, version 5.2 (Tamura et al., 2011). Bootstrap values were estimated (with 1000 replicates) to assess the relative support for each branch, and bootstrap values were labeled with cutoff = 50. Promoter prediction was operated by PlantPAN2^2^. Sequence data from this article can be found in Supplementary Table S2.

### Quantitative RT-PCR (qRT-PCR)

The expression levels of *HpLcyB*, *HpLcyE*, *HpPsy*, and *HpChyB* in algal cells under environmental stresses were determined by quantitative RT-PCR (qRT-PCR). qRT-PCR was performed with a 7300 Real-Time PCR System (Applied Biosystems, United States) using the PrimeScript^®^ RT reagent Kit With gDNA Eraser (Perfect Real Time) (which supplies RNase-free DNase I to remove any coisolated genomic DNA) and the SYBR^®^ *Premix Ex Taq*^TM^ II Kit (Tli RNaseH Plus) (TaKaRa, China). The reaction mix contained 4 μL of cDNA, 0.5 μL of forward and reverse primer mix (20 μM each), 1 μL of 50× ROX Reference Dye, and 25 μL of 2× TaKaRa SYBR Green PCR mix in a final volume of 50 μL. All reactions were set up in triplicate, and every sample was replicated in parallel three times to ensure statistical relevance. The following standard thermal conditions were used for all PCRs: 30 s at 95°C, and then 40 cycles of 30 s at 95°C and 31 s at 60°C. Primer specificity was confirmed by RT-PCR amplification before qRT-PCR reaction, which produced single amplicons of the expected size for each primer set; these amplicons were sequenced to finally validate their specific amplification. The specificity of qRT-PCR reaction was monitored by the presence of dissociation curves with single peaks. Amplicon dissociation curves were obtained after cycle 40 with default settings recommended by the instrument. Data were analyzed using the SDS software (Applied Biosystems, United States). All quantifications were normalized to the amount of the house-keeping gene β-actin as an internal control (Huang et al., 2006; Vidhyavathi et al., 2008). The primers used for qRT-PCR are listed in Supplementary Table S1.

### Plasmid Construction and Transformation

The plasmid pACCRT-EIB was used as a control or a backbone for enzymatic assays and substrate specificity, which harbors *crtE*, *crtI*, and *crtB* from *Pantoea ananatis* encoding GGPP synthase (GGPPS), phytoene desaturase (PDS), and phytoene synthase (PSY), respectively, and confers accumulation of lycopene to *E. coli* strains (Misawa et al., 1990). The open reading frames (ORFs) of *HpLcyB* and *HpLcyE* genes were inserted into the *Hind*III site of the plasmid pACCRT-EIB to produce plasmids pACCRT-EIB-B and pACCRT-EIB-E, respectively. The ORFs of *Rhodobacter capsulatus crtD* (GenBank: J04969.1) and *Synechococcus elongatus* PCC 7942 *Pds* (GenBank: X55289) were then inserted into the *Nde*I site of the plasmids pACCRT-EIB-B and pACCRT-EIB-E to produce plasmids pACCRT-EIBneurB and pACCRT-EIBneurE, pACCRT-EIBzetaB and pACCRT-EIBzetaE, respectively. These insertions inactivated the *crtI* gene in the pACCRT-EIB backbone, and supplied neurosporene and ζ-carotene by the *R. capsulatus crtD* (Bartley and Scolnik, 1989) and *S. elongatus Pds* genes (Chamovitz et al., 1991), respectively, for substrate specificity assays. The ORFs of *HpLcyE* and *HpLcyB* genes were fused and inserted into the *Bam*HI and *Hind*III sites of the expression vector pET-32a-c(+) to produce plasmid pET-32a-LCYE-B. For expression of His-tagged cyclase enzymes, the truncated ORFs without the predicted chloroplast transit peptides of both cyclases were inserted into the *Bam*HI and *Hind*III sites of the expression vector pET-32a-c(+) to produce plasmids pET-32a-LCYB and pET-32a-LCYE, respectively. The codons of HpLCYE were optimized to those of *E. coli* for all functional assays here. All insertions were carried out using the In-Fusion^®^ HD Cloning Kit (Clontech, United States) according to the user manual. All cloning primers were designed following the instructions of the kit.

The plasmids pACCRT-EIB, pACCRT-EIB-E, and pACCRT-EIB-B were transformed into *E. coli* strain DH5α, respectively, for enzymatic activity assays. For substrate specificity assays, the plasmids pACCRT-EIBneurB, pACCRT-EIBneurE, pACCRT-EIBzetaB, and pACCRT-EIBzetaE were transformed, respectively; the plasmids pACCRT-EIB and pET-32a-LCYE-B were co-transformed into *E. coli* strain DH5α. The plasmids pET-32a-LCYB and pET-32a-LCYE were transformed into *E. coli* strain BL21 (DE3) for the production of His-tagged cyclase enzymes. The empty vector pET-32a-c(+), as a control, was also transformed into *E. coli* strains DH5α and BL21 (DE3), respectively.

### Heterologous Expression of Cyclases in *E. coli*

The *E. coli* BL 21 (DE3) cells containing the plasmid pET-32a-LCYB or pET-32a-LCYE were grown to an OD_600_ = 0.6 and induced with 1 mM IPTG for 3 h at 30°C. Cells were pelleted by centrifugation at the speed of 12,000 *g* for 2 min at 4°C. Enzyme extracts were then prepared by the Ni-NTA Spin Kit (QIAGEN, German) according to the user protocol, and were eluted using 300 μL of PBS (50 mM NaH_2_PO_4_, 300 mM NaCl, pH 7.0) containing 500 mM imidazole. The PBS buffer was then exchanged to Tris-HCl buffer (0.1 M Tris-HCl, pH 8.0, 5 mM DTT, 1 mM EDTA) by using the Zeba Spin Desalting Columns and Plates, 7K MWCO (Thermo Fisher Scientific, United States). Protein extracts were quantitated at 25°C by the TaKaRa BCA Protein Assay Kit (TaKaRa, China). All steps were carried out at 4°C unless otherwise stated.

### Enzymatic Assays

The enzymatic activities of HpLCYB and HpLCYE was determined using 50 mM γ-carotene as substrate and 1 μg of purified heterologously expressed protein in 200 μL of 0.1 M Tris-HCl at pH 7.0 with 10% v/v glycerol, 5 mM DTT and 1 mM EDTA, respectively. After incubation under shaking at the speed of 180 rpm in dark for 1 h at 30°C, the reactions were immediately terminated by liquid nitrogen. Then the reaction mixtures were centrifuged at 15,000 *g* for 5 min at 4°C. Supernatants were collected and extracted with acetone, 5 μL of which was subjected to UPLC analysis. To each individual enzyme assay, negative controls with protein extracts from *E. coli* transformed with the empty plasmid pET-32a-c(+) were included.

### Statistical Analysis

The data were processed by one-way analysis of variance using SPSS version 13.0 (SPSS, United States). Summary statistics were expressed as means ± standard deviations (SD). In all statistical analyses, *P* < 0.05 was considered statistically significant.

## Results

### Carotenoid Profiles of *H. pluvialis* under Environmental Stresses Indicate the *In Vivo* Activity of a ε Cyclase

**FIGURE 2:**
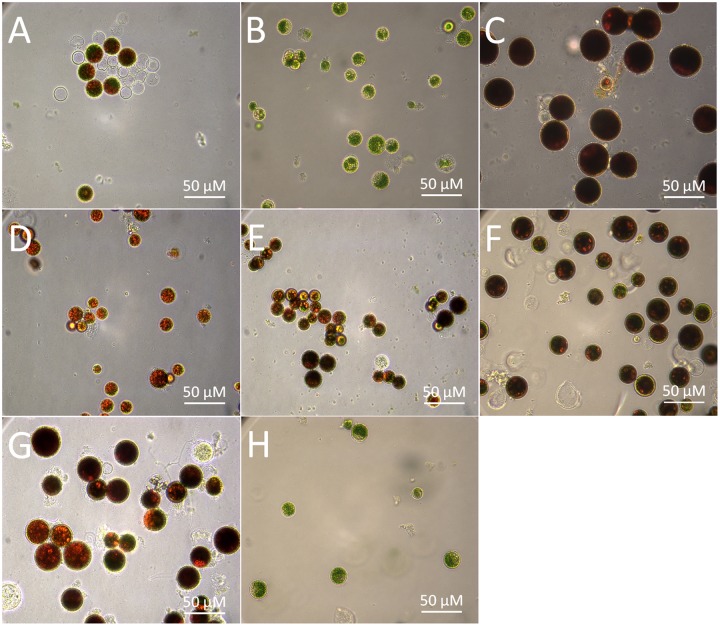
Morphological changes of the *H. pluvialis* cells imposed by different environmental stresses. **(A)** Control; **(B)** dark; **(C)** high light; **(D)** N deficiency (N^-^); **(E)** P deficiency (P^-^); **(F)** NaCl; **(G)** oxidative stress (FeSO_4_+SA); **(H)** green vegetable cells.

Stress-induced synthesis of secondary astaxanthin in *H. pluvialis* is accompanied by remarkable morphological and biochemical transformation from green vegetable cells into inert red cysts (Boussiba, 2000). In this study, green vegetable cells at the late logarithmic phase (**Figure 2H**) were imposed to induce astaxanthin accumulation by high light, nutrient starvation (N or P deficiency), high salinity, and oxidative stress (FeSO_4_+SA), which are often defined as inductive conditions (Boussiba, 2000). As shown by **Figure 2**, most cells transformed into large red cysts (aplanospores) with red globules in the center of cells where astaxanthin was synthesized after cultivation for 30 days. High light, NaCl, and FeSO_4_+SA were the three most inductive stresses that forced cells to enlarge size and form fully mature cysts (aplanospores) (**Figures 2C,F,G**).

The morphological changes diversely responded to environmental stresses and corresponded to the level of secondary carotenoids, mainly astaxanthin (**Table 1**). Concomitant with the morphological changes in cells imposed by high light, NaCl, and FeSO_4_+SA was enormous astaxanthin accumulated, to ∼4.88, 2.40, and 1.92 mg g^-1^ DCW of total astaxanthin, respectively. The content of astaxanthin between the control and nutrient starvation did not vary significantly. This unconspicuous difference is likely to result from the prolonged cultivation period (30 days) used, which was also indicated by the similarity in morphology, i.e., partly mature cysts developed in the control (**Figure 2A**), N deficiency (**Figure 2D**), and P deficiency (**Figure 2E**). No secondary carotenoids was detected in the dark-treated cells which grew slowly into a small green vegetable figure (**Figure 2B**).

**Table 1:** Carotenoid profiles of *Haematococcus pluvialis* treated by environmental stresses.

	Contents of analytes (mg g^-1^ DCW, *n* = 3)						
	
	CK	HL	Dark	*N^-^*	*P^-^*	FeSO_4_+SA	NaCl
*Trans*-asta	0.87 ± 0.05	2.25 ± 0.10	n.d.	0.25 ± 0.04	0.36 ± 0.06	0.99 ± 0.10	1.69 ± 0.53
*Cis*-asta	0.36 ± 0.04	2.63 ± 0.12	n.d.	0.67 ± 0.09	0.82 ± 0.09	0.93 ± 0.08	0.71 ± 0.07
Total asta	1.23	4.88	n.d.	0.92	1.18	1.92	2.4
Lutein	1.744 ± 0.21	0.35 ± 0.09	0.72 ± 0.11	0.23 ± 0.07	0.522 ± 0.12	0.24 ± 0.11	0.45 ± 0.10
Cantha	0.14 ± 0.02	0.19 ± 0.03	n.d.	0.14 ± 0.03	0.09 ± 0.00	0.07 ± 0.00	0.11 ± 0.00
Lyc	0.67 ± 0.18	0.98 ± 0.71	n.d.	n.d.	n.d.	0.46 ± 0.06	0.68 ± 0.32
δ-car	n.d.	n.d.	n.d.	n.d.	n.d.	n.d.	n.d.
α-car	n.d.	n.d.	n.d.	n.d.	n.d.	n.d.	0.08 ± 0.00
β-car	0.88 ± 0.08	0.33 ± 0.03	0.72 ± 00.07	0.15 ± 0.03	0.28 ± 0.03	0.24 ± 0.03	0.55 ± 0.10


*Data are expressed as dry cell weight (DCW) of carotenoids, and each value is the mean ± SD for three experiments (*n* = 3). Asta, astaxanthin; cantha, canthaxanthin; lyc, lycopene; δ-car, δ-carotene; α-car, α-carotene; β-car, β-carotene; CK, control; HL, high light; N^-^, N deficient; P^-,^ P deficient; n.d., not detected.*

In contrast, the content of lycopene, β-carotene, and lutein was relatively low (**Table 1**). An overall decrease in β-carotene and lutein was observed in the stressed cells. Lycopene was not detected in cells stressed by dark and nutrient starvation; but, compared to the control, a slight increase was induced by high light. N deficiency resulted in the most declined levels of these carotenoids. Under the control condition, lutein was the major carotenoid accumulated and accounted for ∼50% of total carotenoids, implying that (1) a yet to be identified LCYE exists; or that (2) the single LYCB can also make epsilon rings; or that 3) a completely novel LCYE exists and evolved that has no homology to known LCYE. Remarkably, however, trace α-carotene (∼0.08 mg g^-1^ DCW) was determined in cells treated by NaCl, which strongly indicated the *in vivo* activity of a LCYE enzyme that has not yet to be functionally identified, despite the fact that no direct product (δ-carotene) of the enzyme was detected.

### Sequence Isolation and Functional Complementation of the *H. pluvialis* Cyclases

**FIGURE 3:**
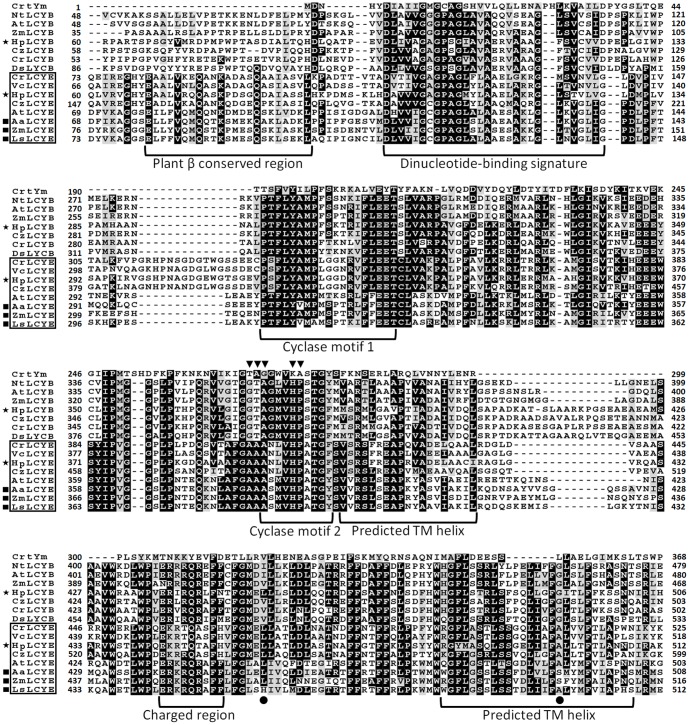
Multiple secondary domains in cyclases. Highly structural resemblance between the β and ε cyclases suggests that they may have a similar mechanism of action (Ronen et al., 1999). The regulatory motif conserved in all β bicyclases are highlighted by arrowheads. The two residues varying between ε mono- and bicyclases are highlighted by filled cycles.

Sequence isolation revealed that the *HpLcyE* and *HpLcyB* genes are 2439 and 2375 bp in length encoding proteins of 575 aa and 568 aa, respectively, which share the highest similarity with algal cyclases, e.g., 59–68% for LCYEs and 67–71% for LCYBs, correspondingly. Alignment of the amino acid sequences of HpLCYE and HpLCYB with plant lycopene cyclases demonstrated a marked sequence conservation, 36% identity among and between LCYEs and LCYBs in plant and algae (**Figure 3**). Both HpLCYB and HpLCYE contain a dinucleotide-binding motif that is found in all lycopene cyclases and is thought to bind FAD; and a plant β conserved region exclusively found in plant-type cyclases (CrtL) rather than bacterial CrtYm, which is thought to be crucial in specific interaction between the cyclase and components of the membrane (Hugueney et al., 1995). Three well-conserved regions, cyclase motif 1, 2, and charged region, potentially involved in substrate binding and catalysis (Cunningham et al., 1996), were also found.

**FIGURE 4:**
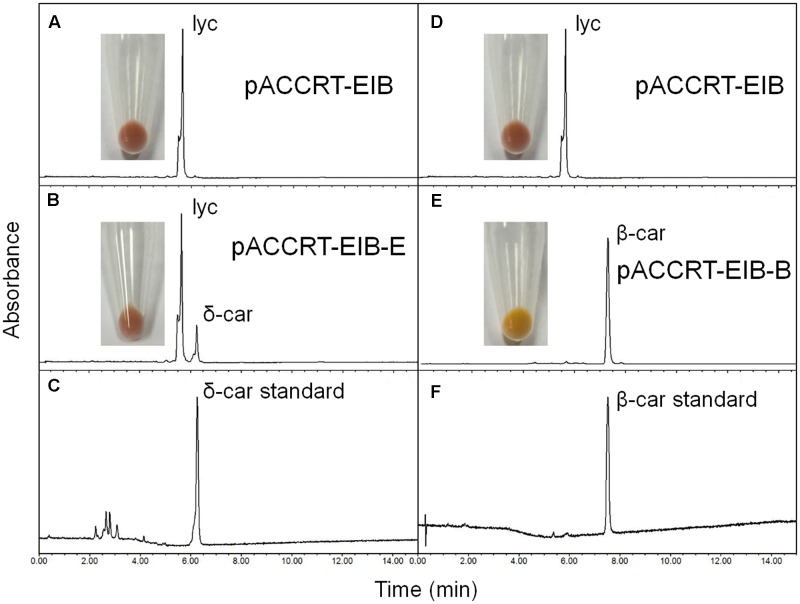
Functional complementation of the *H. pluvialis* cyclases. Note that the activity of HpLCYE was relatively low, as a large amount of lycopene substrate had been left, which led to an unobvious color change in between the *E. coli* cultures carrying pACCRT-EIB and pACCRT-EIB-E, respectively; and that HpLCYE solely produced monocyclic δ-carotene, while HpLCYB produced bicyclic β-carotene, indicating the corresponding monocyclase or bicyclase activity. Lyc, lycopene; δ-car, δ-carotene; β-car, β-carotene. **(A,D)** Lycopene synthesized by the *E. coli* strain carrying the plasmid pACCRT-EIB; **(B)** δ-carotene synthesized by adding HpLCYE to the lycopene-producing strain (pACCRT-EIB-E); **(C)** δ-carotene standard; **(E)** β-carotene synthesized by adding HpLCYB to the lycopene-producing strain (pACCRT-EIB-B); **(F)** β-carotene standard.

The activity of the *H. pluvialis* cyclases was validated in *E. coli* by supplying lycopene as substrate (**Figure 4** and **Table 2**). *E. coli* cells carrying the plasmid pACCRT-EIB accumulated lycopene (**Figures 4A,D**). Addition of one or more ε and/or β cyclic end groups to pink lycopene would result in products that are orange-yellow or yellow in color. As **Figure 4E** demonstrated, the addition of HpLCYB to the lycopene-accumulating cells led to an obvious color change from pink to yellow in the cultures. The change in color depended on the ability of HpLCYB to cyclize both ends of lycopene to produce β-carotene, presumably through γ-carotene intermediate. While the addition of HpLCYE cyclized only one end of lycopene to synthesize δ-carotene, rather than both ends to generate ε-carotene (**Figure 4B**). No γ-carotene intermediate was detected in the assay for HpLCYB, probably due to its strong bicyclase activity. In contrast, the activity of HpLCYE was relatively low, resulting in a large amount of lycopene substrate being left and an indistinctive change in color in the cultures (**Figure 4B**). The functional complementation tests confirmed the authenticity of the isolated *HpLcyE* and *HpLcyB* encoding a ε monocyclase and a β bicyclase, respectively.

**Table 2:** Product profiles of the *H. pluvialis* cyclases and site-directed mutants expressed in *E. coli*.

	Percentage of total carotenoids (*n* = 3)								
	
	lyc	δ-car	γ-car	ε-car	α-car	β-car	neur	α-zeacar	β-zeacar
EIB-E	67.89 ± 2	32.11 ± 3	n.d.	n.d.	n.d.	n.d.	n.d.	n.d.	n.d.
EIB-B	1.08 ± 1	n.d.	n.d.	n.d.	n.d.	98.92 ± 4	n.d.	n.d.	n.d.
EIBneurE	3.88 ± 2.5	n.d.	n.d.	n.d.	n.d.	n.d.	93.6 ± 1	2.53 ± 2	n.d.
EIBneurB	n.d.	n.d.	n.d.	n.d.	n.d.	n.d.	n.d.	n.d.	100 ± 3
EIB+EB	12.36 ± 2	2.65 ± 1	5.22 ± 0.5	n.d.	17.04 ± 2	62.73 ± 3	n.d.	n.d.	n.d.


*Data are expressed as dry cell weight (DCW) percentages of total carotenoids in the *E. coli* strains, and each value is the mean ± *SD* for three experiments (*n* = 3). Lyc, lycopene; δ-car, δ-carotene; γ-car, γ-carotene; ε-car, ε-carotene; α-car, α-carotene; β-car, β-carotene; neur, neurosporene; α-zeacar, α-zeacarotene; β-zeacar, β-zeacarotene; n.d., not detected.*

### Substrate Specificity of the *H. pluvialis* Cyclases

**FIGURE 5:**
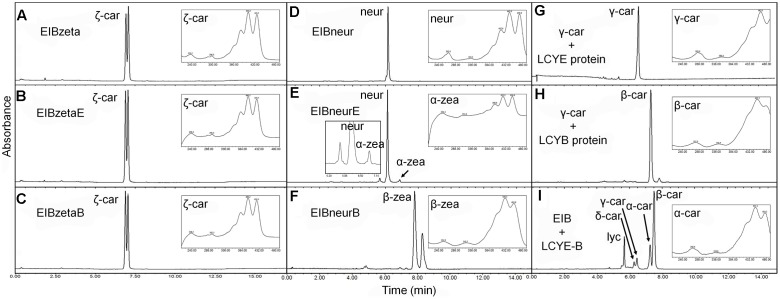
Substrate specificity of the *H. pluvialis* cyclases. **(A**–**C)** Both HpLCYE and HpLCYB failed to utilize ζ-carotene; **(D**–**F)**, both HpLCYE and HpLCYB catalyzed neurosporene to produce α- **(E)** and β-zeacarotene **(F)**, respectively; **(G)** HpLCYE failed to catalyzed γ-carotene; **(H)** while HpLCYB was able to catalyze δ-carotene to produce β-carotene; **(I)** when both cyclases were co-introduced into the lycopene-accumulating *E. coli* strain, complex product profile was generated, with β-carotene and α-carotene as the major products. Both HpLCYB and HpLCYE require substrates desaturated at the 7-8 or 7’-8’ carbon-carbon double bond. Note that lycopene must be cyclized exclusively following the order of HpLCYE to HpLCYB to synthesize α-carotene. Absorption spectra of representative carotenoids shown in boxes are embedded into each panel, correspondingly. In **(E)**, an enlarged chromatogram is also present in box at the left bottom. Lyc, lycopene; ζ-car, ζ-carotene; neur, neurosporene; α-zea, α-zeacarotene; β-zea, β-zeacarotene; δ-car, δ-carotene; γ-car, γ-carotene; α-car, α-carotene; β-car, β-carotene.

Substrate specificity was determined by the *E. coli* strains harboring the plasmids capable of accumulating various carotenoids such as pACCRT-EIB for lycopene, pACCRT-EIBzeta for ζ-carotene, and pACCRT-EIBneur for neurosporene, or by enzymatic assays using purified HpLCYE and HpLCYB proteins (**Figure 5** and **Table 2**). The UPLC elution profiles and absorption spectra of some of these experiments are illustrated in **Figure 5**. The empty expression vector pET-32a-c(+) was used as a control, which did not affect the composition of pigments accumulated by the carotenoid-accumulating *E. coli* strains (data not shown).

Both HpLCYE and HpLCYB did not change the composition of pigments accumulated in their hosts carrying pACCRT-EIBzeta, respectively, as indistinguishable elution profiles and corresponding absorption spectra were observed (**Figures 5B,C**). Similar to the control (**Figure 5A**), two peaks of ζ-carotene isomers were observed in these strains, separately. However, when HpLCYE and HpLCYB were, respectively, co-introduced into the *E. coli* cells harboring pACCRT-EIBneur, different products were generated, i.e., neurosporene produced by pACCRT-EIBneur was probably transformed into α-, and β-zeacarotene, respectively. **Figures 5D–F** showed the shifts of carotenoids composition.

As discussed above, HpLCYB could cyclize both ends of lycopene to form β-carotene (**Figure 4E**); while HpLCYE catalyzed the cyclization of only one end of lycopene to form δ-carotene (**Figure 4B**). Meanwhile, neither γ- nor ε-carotene was detected in the algal cell extracts by UPLC (**Table 1** and **Supplementary Figure S1**). Here, we speculated that γ-carotene intermediate was instantaneously transformed into β-carotene by the robust activity of HpLCYB, making it difficult to be detected; and that HpLCYE seems to be a monocyclase that merely catalyzed the cyclization of one end of lycopene in the current assays.

To further confirm the monocyclase activity of HpLCYE and the sequence of cyclization for α-carotene biosynthesis, we 1) operated *in vitro* enzymatic assays using γ-carotene as substrate and purified cyclase proteins heterologously expressed in *E. coli* cells; and 2) simultaneously expressed HpLCYE and HpLCYB in the lycopene-accumulating *E. coli* cells (carrying pACCRT-EIB). Enzymatic assays showed that HpLCYE failed to cyclize γ-carotene to form α-carotene (**Figure 5G**); while HpLCYB further cyclized γ-carotene to form β-carotene (**Figure 5H**). When simultaneously expressed in the lycopene-accumulating host, HpLCYE and HpLCYB competitively catalyzed lycopene to form complex mixture of carotenoid pigments (**Figure 5I**). The predominant was β-carotene, followed by a considerable amount of α-carotene. A modicum of δ- and γ-carotene were also detected. Thus, the substantial α-carotene was expected to be synthesized synergistically and sequentially by the cyclases through a successive cyclization pattern that HpLCYE cyclized one end of lycopene to form monocyclic δ-carotene antecedently, followed by subsequently cyclizing the other end by HpLCYB, to form the final bicyclic product.

### Transcriptional Expression of the *H. pluvialis* Cyclases

**FIGURE 6:**
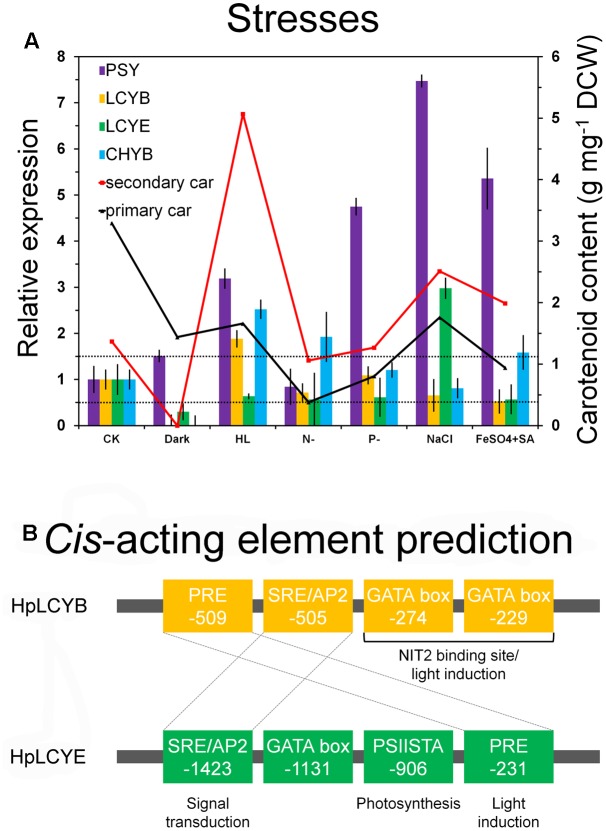
Expression pattern and bioinformatic analysis of promoter regions of the *H. pluvialis* cyclases. Differential expression of the *H. pluvialis* cyclases correlated with the synthesis of primary and secondary carotenoids **(A)**, and potential regulatory elements in promoter regions, respectively **(B)**. The interval defined between 0.5 and 1.5-folds as no change is illustrated by dashed lines in the expression pattern study. Regulatory elements shared by HpLCYB and HpLCYE are crossed by dashed lines in the bioinformatic analysis.

The steady-state levels of the mRNA of *HpLcyE* and *HpLcyB*, together with the rate-limiting *HpPsy* and astaxanthin-synthesizing *HpChyB*, were determined by qRT-PCR. Compared to the control, transcripts between 0.5 and 1.5-folds were set up as no change; 0.5-fold or less as decrease; 1.5-folds or greater as increase. *HpPsy* and *HpChyB* are the most determined regulators for astaxanthin biosynthesis. *HpPsy* was up-regulated by almost all stresses, as much as ∼1.5 to 7-folds of that of the control, except N deficient that did not apparently change its transcription (**Figure 6A**). NaCl, followed by FeSO_4_+SA which induced more than fivefolds of the *HpPsy* transcripts, is the strongest stress that significantly promoted *HpPsy* transcription to more than sevenfolds. Dark could slightly induced *HpPsy* transcription to ∼1.5-folds. Compared to *HpPsy*, *HpChyB*, and *HpLcyB* were induced exclusively by high light, more than 2.5-folds for *HpChyB* and ∼1.9-folds for *HpLcyB*, respectively. Moderate increase in the *HpChyB* transcripts was also observed in N deficiency and FeSO_4_+SA, ∼1.9 and 1.5-folds, respectively. In contrast, dark completely shut down the expression of *HpChyB*.

Whereas, expression of *HpLcyE* and *HpLcyB* did not changed appreciably in response to most stresses (**Figure 6A**). Dark considerably reduced the *HpLcyE* transcripts (∼0.3-folds) and almost switched off the expression of *HpLcyB*. While NaCl evidently up-regulated the *HpLcyE* transcripts by ∼3-folds, though it did not affect *HpLcyB*. Overall, the expression of cyclases correlated with the profiles of primary carotenoids (**Supplementary Figure S1**), specifically, trace α-carotene determined exclusively in NaCl stress; and mildly decreased β-carotene and lutein, compared to the control (**Table 1**).

## Discussion

### The Physiological Roles of Primary and Secondary Carotenoids under Environmental Stresses

According to the biological functions, carotenoids are classified into primary and secondary carotenoids. Primary carotenoids are structural and functional components of the cellular photosynthetic apparatus and therefore are essential for survival (Niyogi et al., 1997). Generally, they are non-inductive by environmental stresses. Secondary carotenoids such as canthaxanthin and astaxanthin are induced to a large amount via carotenogenesis only when cells are exposed to environmental stresses (Jin et al., 2003).

It is suggested that stress-induced synthesis of secondary carotenoids is mediated by ROS (Boussiba, 2000). Excessive light irradiation leads to photoinhibition by the over-reduction of plastoquinone in photosystem II (PSII) and the formation of ROS in the reaction center of PSII (Wang et al., 2003). Nutrient starvation, high salinity, and oxidative stress also lead to the formation of ROS by limiting CO_2_ fixation and generating excessive reducing power in photosynthesis (Boussiba, 2000). Accumulation of secondary carotenoids is a protective strategy against ROS. Massively induced astaxanthin (**Table 1**) acts as sunscreen by dissipating excessive light energy and shielding the photosynthetic apparatus, and as antioxidant and physicochemical barrier against photodynamic damage by ROS (Wang et al., 2003).

Lycopene, β-carotene, and lutein seemed to be non-inductive (**Table 1** and **Supplementary Figure S1**). Therefore, they are regarded as primary carotenoids which involve in photosynthesis as accessory light-harvesting pigments bound with chlorophyll within the light-harvesting complexes (LHC) (Niyogi et al., 1997). The decrease in primary carotenoids probably resulted from a strong inhibition of photosynthetic activity in *H. pluvialis* cells, which simultaneously decreased the content of bound chlorophylls (Bar et al., 1995; Masojídek et al., 2000; Vidhyavathi et al., 2008). Evidences that expression of chlorophyll biosynthesis and LHC related genes was significantly inhibited by high light and nutrient starvation (Kim et al., 2011), and that primary carotenoids and chlorophylls were the dominant pigments in green motile cells at non-inductive condition support this speculation (Vidhyavathi et al., 2008; Gwak et al., 2014).

The role of β-carotene as primary carotenoids is species-specific. Generally, primary carotenoids are located in the chloroplast, while secondary carotenoids within globular regions of the cytoplasm. In *H. pluvialis*, two types of β-carotene have been found: one is co-located with chlorophylls, while the other with astaxanthin in the cytosol (Collins et al., 2011). The former belongs to primary carotenoids, the latter to secondary carotenoids. In this study, the detected β-carotene is likely to be mainly primary carotenoids because its content was not induced by environmental stresses (**Table 1**). Whereas, in *Dunaliella bardawil* and *Trentepohlia aurea*, β-carotene was induced by light stress and should be considered principally as secondary carotenoids (Bar et al., 1995). It should be noted that we did not exclude any secondary β-carotene, which seemed to be at a very low level due to the depletion by downstream reactions for astaxanthin biosynthesis. Such might be the case in α-carotene.

### Molecular Evolution of LCYE

The complex secondary structure of cyclases (**Figure 3**) might be recruited from multiple protein families for catalysis exchanges that are particularly evident in the coevolutionary relationships displayed by some carotenoid biosynthetic lineages with other biochemical structures, e.g., proteorhodopsins and the photosynthetic reaction center (Klassen, 2010; Cui et al., 2011). The unique structural coevolution and high sequence conservation between the β- and ε-cyclases suggested that the two types of enzymes have a similar mechanism of action (Ronen et al., 1999), both are proposed to catalyze lycopene in reactions through a common carbonium ion intermediate (Frank and Cogdell, 1996).

**FIGURE 7:**
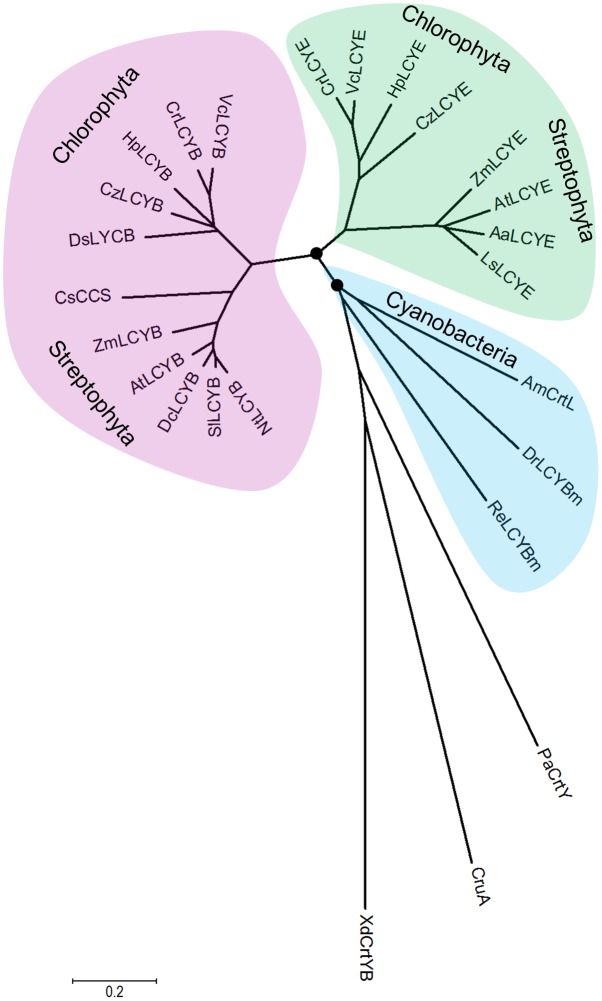
Molecular evolutionary relationships of the *H. pluvialis* cyclases by the Neighbor-Joining method. The nodes of gene duplication during evolution are dotted. CCS: capsanthin-capsorubin synthase.

The overall sequence conservation and highly structural resemblance between the β and ε cyclases (**Figure 3**), as well as similar mechanism of catalysis (Frank and Cogdell, 1996), suggest that plant-type cyclases have evolved from a common ancestor. To investigate the molecular evolution of the cyclases, a phylogenetic tree (**Supplementary Figure S2**) was constructed by using the Neighbor-Joining method (Saitou and Nei, 1987). To validate the robustness of the analysis, a Maximun-Likelihood tree (Jones et al., 1992) was also constructed (**Supplementary Figure S3**). The two tree topologies match perfectly with each other, and the high Neighbor-Joining bootstrap values coincide with the high branch support values, suggesting that the phylogeny of the cyclases is robust in different tree reconstruction methods. The radiation mode of the Neighbor-Joining tree shown in **Figure 7** illustrated that all plant and algal cyclases display a close evolutionary position to the cyanobacterial CrtL (AmCrtL). It is likely that algal, plant, and cyanobacterial cyclases have evolved from a common plant-type LCYB that appeared first in cyanobacteria. Plant and algal LCYEs clustered into a group which was encompassed by the plant-type LCYBs, suggested that LCYEs have evolved from LCYBs maybe through gene duplication, particularly in algae where the light harvesting antenna complexes of protein-bound chlorophylls and xanthophylls first developed, and where lutein and other ε-ring xanthophylls are the predominant carotenoids (Ronen et al., 1999).

Comparative genomic and phylogenetic analyses of the algal cyclases revealed that several green algae retained only one type of cyclase, either LCYB or LCYE, owing to gene lost in evolution (Cui et al., 2011). These green algae include *H. pluvialis* and *D. salina* that were computationally identified to contain only LCYB (Cui et al., 2011). The isolation and functional complementation of the missing HpLCYE, along with HpLCYB, renew our knowledge on that the co-existence of the two types of cyclase suggests an important regulatory role in controlling linear substrates into branch points leading to β-ring astaxanthin (secondary carotenoids) or ε-ring lutein (primary carotenoids). The former is believed to be sensitive to and copes with unfavorable circumstances, for example, as antioxidant against ROS aroused by environmental stresses (Boussiba, 2000; Wang et al., 2003); while the latter is irreplaceable for photosynthesis and survival as structural pigment bound with chlorophylls in LHC (Niyogi et al., 1997).

### Differential Regulation of the *H. pluvialis* Cyclases by *Cis*-Acting Elements to Control Metabolic Flux Towards Astaxanthin Biosynthesis in Cysts

Secondary carotenoids in the *H. pluvialis* cells forced by environmental stresses was measured to correlate with the transcriptional expression of *HpPsy* and *HpChyB* (**Figure 6A**). High light orchestrated the expression of *HpPsy* and *HpChyB* to maximize the synthesis of secondary carotenoids, ∼5.07 mg g^-1^ DCW, while NaCl and FeSO_4_+SA moderately enhanced their synthesis to ∼2.51 and 1.99 mg g^-1^ DCW, respectively, compared to the control (∼1.37 mg g^-1^ DCW). No secondary carotenoids was detected in the dark-treated cells. Though *HpLcyB* was also induced by high light, the content of β-carotene did not increase. As discussed above, secondary β-carotene synthesized by HpLCYB might be exhausted by downstream enzymes including HpCHYB for astaxanthin biosynthesis, leaving the primary β-carotene being detected. The generally unchanged expression of *HpLcyE* and the small amount of products suggested that metabolic flux is largely shunted into the synthesis of secondary astaxanthin under environmental stresses. Actually, a significant decrease in the expression of photosynthesis-related genes, i.e., chlorophyll biosynthesis and LHC related genes, in astaxanthin-inducing conditions such as high light and nutrient starvation did correlate with the accumulation of secondary astaxanthin in this alga (Eom et al., 2006; Kim et al., 2011; Gwak et al., 2014).

At the branch points, HpLCYE and HpLCYB are differentially regulated at the transcriptional (**Figure 6A**) and metabolic levels (**Table 1**) to rapidly adapt to environmental stresses by elaborately apportioning metabolic flux into the biosynthesis of primary and secondary carotenoids, respectively. At the transcriptional level, differentially regulating key genes in carotenoid biosynthesis often originates from the diversity of *cis*-acting elements in the promoter regions (Lao et al., 2011; Lao et al., 2014). An example is the carotenogenesis genes in *D. bardawil*, which were differentially inducted by salt stress (Lao et al., 2014). Bioinformatic comparison of the promoter regions of carotenogenesis genes would find distinct regulatory sequence architectures that might be responsible for the variant inductions (Lao et al., 2014).

Consequently, bioinformatics analysis of promoter regions against the genome of *C. reinhardtii* was carried out between the promoters of *HpLcyB* and *HpLcyE* (**Figure 6B**). Some *cis*-acting elements potentially involved in light regulation were found to scatter throughout the *HpLcyB* promoter: (1) a tandem GATA boxes with an inter-element spacing of 37 bp that resembles the light-regulated NIT2 binding site (Chiang and Marzluf, 1994; Rastogi et al., 1997); and (2) consensus sequences that resembles the plastid response element (PRE) involved in the light induction of the *C. reinhardtii* HSP70A (von Gromoff et al., 2006). Currently, only the promoter of *Bkt1* in carotenoids biosynthesis had been characterized in *H. pluvialis* before the present study. This promoter contains serval light-regulated element candidates that are likely to confer light induction of the reporter gene in *C. reinhardtii* (Wang et al., 2012, 2016). As *HpLcyB* was significantly induced by light (**Figure 6A**), our findings implied a possible link between the transcriptional pattern and the light-regulated element candidates of the *HpLcyB* promoter.

For the *HpLcyE* promoter, no candidate account for the transcriptional pattern was found. But a PSIISTA motif potentially involved in photosynthetic regulation was found (**Figure 6B**). The PSIISTA motif was first found in the 5′-UTR of the chloroplast *psbD* mRNA of *C. reinhardtii*, which functioned as a mRNA stabilizer for *psdD* expression (Nickelsen et al., 1999). The *psbD* mRNA of *C. reinhardtii* is one of the most abundant chloroplast transcripts which encodes the PSII reaction center polypeptide D2 (Nickelsen et al., 1999). Two *cis*-acting elements, PRE, and SRE/AP2, are shared by the promoters of *HpLcyB* and *HpLcyE*. As mentioned above, PRE is a light-regulated element, thus we suspended the authentic role of this motif in the *HpLcyE* promoter, which was non-inductive under high light (**Figure 6A**). The SRE/AP2 motif is possible to participate in signal transduction, particular in stress signaling (Chang et al., 2013; Weirauch et al., 2014), considering that *HpLcyB* was induced by high light while *HpLcyE* by salt.

### Mechanism for Control of Cyclic Carotenoid Composition

The ability of HpLCYE to catalyze neurosporene was extremely weak, implying that neurosporene is not an effective substrate (low affinity) for HpLCYE; and/or that the catalytic activity of this protein heterologously expressed in *E. coli* was relatively low under the current conditions. Even for the theoretically most suitable substrate (lycopene), the amount of δ-carotene product was relatively low, yet (**Figure 4B** and **Table 2**). When both cyclases were co-expressed in the lycopene-accumulating *E. coli* cells, HpLCYB exhibited a higher activity than HpLCYE, as predominant β-carotene over α-carotene was observed in the assay (**Figure 5I**). It is unlikely that the abundance of HpLCYB enzyme was higher than that of HpLCYE in *E. coli* cells, because we used the same ribosome binding site for them, rendering almost equal strength of translation initiation under a single T7 promoter in the pET-32a-c(+) backbone. According to Cunningham et al (Cunningham et al., 1996), three factors could affect the catalytic activity: (1) the chloroplast transit peptides of the plastid-localized cyclases were inappropriately cleaved; (2) the enzymes heterologously expressed in *E. coli* were impertinently folded, processed, and modified as they might be in *H. pluvialis*; and (3) the environment in *E. coli* might not fit that in *H. pluvialis*, for example lipid composition of the membrane where these enzymes resided. Nonetheless, we could not rule out the possibility that the activity of HpLCYE is inherently low, because no direct product (δ-carotene) of the enzyme was detected in *H. pluvialis* cells (**Table 1**). Of course, we should not overlook that most δ-carotene had been used up by downstream reactions for xanthophylls biosynthesis, as was possible for β- and α-carotene; and that the conditions used to stimulate the algal cells were not proper for the enzyme.

Thought the activity of HpLCYE was relatively low, an appreciable amount of γ- and δ-carotene was synthesized when HpLCYE was added to compete for lycopene substrate with HpLCYB (**Figure 5I**). The addition of HpLCYE seemed to lower the bicyclase activity of HpLCYB.

**FIGURE 8:**
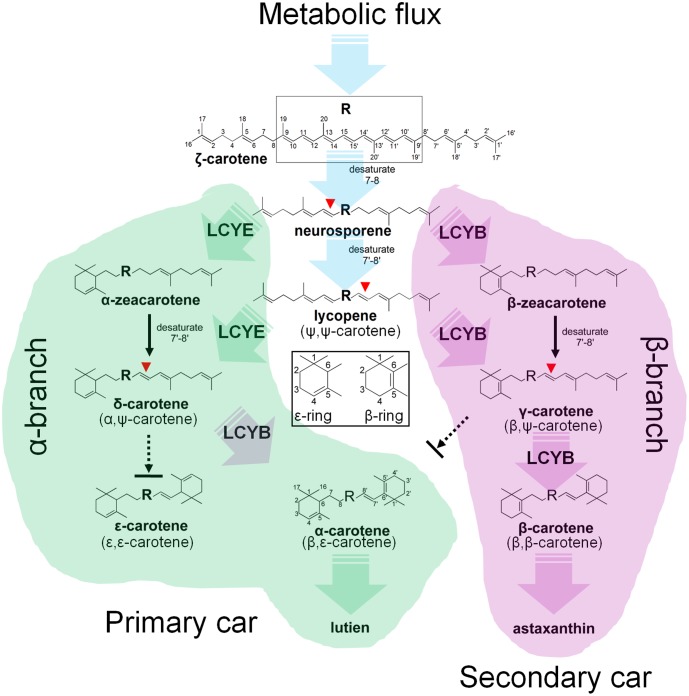
Mechanism of the *H. pluvialis* cyclases for control of cyclic carotenoid composition. Cyclization of acyclic lycopene desaturated at the 7–8 and 7′–8′ carbon–carbon double bond marks an important regulatory branch point in carotenoid biosynthesis. HpLCYE competes with HpLCYB to control metabolic flux to the biosynthesis of primary carotenoids; while HpLCYB regulates metabolic flux to secondary carotenoids. A contact between the α- and β-branch is made during the synthesis of α-carotene and its derivatives, e.g., lutein, which requires a successive cyclization by HpLCYE and HpLCYB.

Cyclization of linear lycopene desaturated at the 7–8 and 7′–8′ carbon-carbon double bonds marks a central branch point in the carotenoid biosynthesis pathway in *H. pluvialis* (**Figure 8**), one route known as α-branch leading to the synthesis of primary lutein is directed by the monocyclase HpLCYE; and the other called β-branch leading to the synthesis of secondary astaxanthin is guided by the bicyclase HpLCYB. The two cyclases are more likely to competitively and synergistically, but not independently as is the case in tomato (Ronen et al., 1999), catalyze lycopene to form α-carotene in an exclusively sequential way that ε-cyclization at one end of lycopene is firstly carried out by HpLCYE, then β-cyclization at the other end is fulfilled by HpLCYB. The competition not only occurs in the access of substrates, resulting in more β-ring derivatives (**Figure 5I** and **Table 2**); but also in the ability of cyclizing ring: the inability of HpLCYE to add more than one ε-ring to linear substrates (**Figures 4B**, **5G**) led to an expected low amount of δ-carotene, most of which was further transformed to β, ε-ring derivatives by HpLCYB (**Figure 5I**). While the biosynthesis of primary β, ε-ring derivatives (e.g., lutein) must be fulfilled by the cooperation of both cyclases. Overall, HpLCYE is likely to mainly steer metabolic flux into the biosynthesis of primary carotenoids; and HpLCYB independently controls metabolic flux into the biosynthesis of secondary carotenoids, and offers assistance to HpLCYE for synthesizing primary carotenoids at the same time. Therefore, the relative activity of HpLCYE versus HpLCYB may determine the flow of carotenoids from lycopene to either α-branch or β-branch. The complexity of regulation of the two cyclases, at the transcriptional (**Figure 6**), protein (**Figures 4**, **5**, **8**), and metabolic levels (**Supplementary Figure S1**, **Figure 2**, and **Table 1**) would be a major mechanism for control of cyclic carotenoid composition. Since α-branch starts at the point of ε-cyclization, mainly through δ-carotene biosynthesis, we believe that the regulation of HpLCYE exerts great influence on at least α-branch carotenoids. The identification of the missing HpLCYE supplied a promising route for molecular breeding of cyclic carotenoid biosynthesis for the cosmetics, nutrient and health industries.

## Author Contributions

YL, HJ, and ZC designed the project. YL and HJ performed most of the experimental work. JZ expressed and purified the HpLCYE and HpLCYB proteins. HZ extracted and saponified the pigments. YL and HJ wrote the manuscript.

## Conflict of Interest Statement

The authors declare that the research was conducted in the absence of any commercial or financial relationships that could be construed as a potential conflict of interest.

## Footnotes

1 http://blast.ncbi.nlm.nih.gov/

2 http://plantpan2.itps.ncku.edu.tw/

## Supplementary Material

**FIGURE S1 |** Carotenoid profiles in the *Haematococcus. pluvialis* cells imposed by stresses. **(A–H)**, CK (control), dark, high light, N deficiency (N^-^), P deficiency (P^-^), NaCl, oxidative stress (FeSO_4_+SA), and carotenoids standards.

**FIGURE S2 |** Molecular evolutionary relationships of the *H. pluvialis* cyclases by the Neighbor-Joining method. The orange, green, red, and blue lines highlight LCYBs, LCYEs, CCS, and fungal bi-functional cyclase, respectively. The nodes of gene duplication during evolution are dotted. The bootstrap consensus tree inferred from 1000 replicates is taken to represent the evolutionary history of the taxa analyzed. Branches corresponding to partitions reproduced in less than 50% bootstrap replicates are collapsed. The percentage of replicate trees in which the associated taxa clustered together in the bootstrap test (1000 replicates) are shown above the branches.

**FIGURE S3 |** Molecular evolutionary relationships of the *H. pluvialis* cyclases by the Maximum Likelihood method. The tree with the highest log likelihood (–12149.8237) is shown. The percentage of trees in which the associated taxa clustered together is shown above the branches. Initial tree(s) for the heuristic search were obtained automatically by applying Neighbor-Join and BioNJ algorithms to a matrix of pairwise distances estimated using a JTT model, and then selecting the topology with superior log likelihood value. The tree is drawn to scale, with branch lengths measured in the number of substitutions per site.
