# Supplementary figures and images for: Functional Characterization of a Missing Branch Component in Haematococcus pluvialis for Control of Algal Carotenoid Biosynthesis
Source: Front Plant Sci. 2017 Aug 2;8:1341. doi: 10.3389/fpls.2017.01341 (PMC5539077; doi:10.3389/fpls.2017.01341)

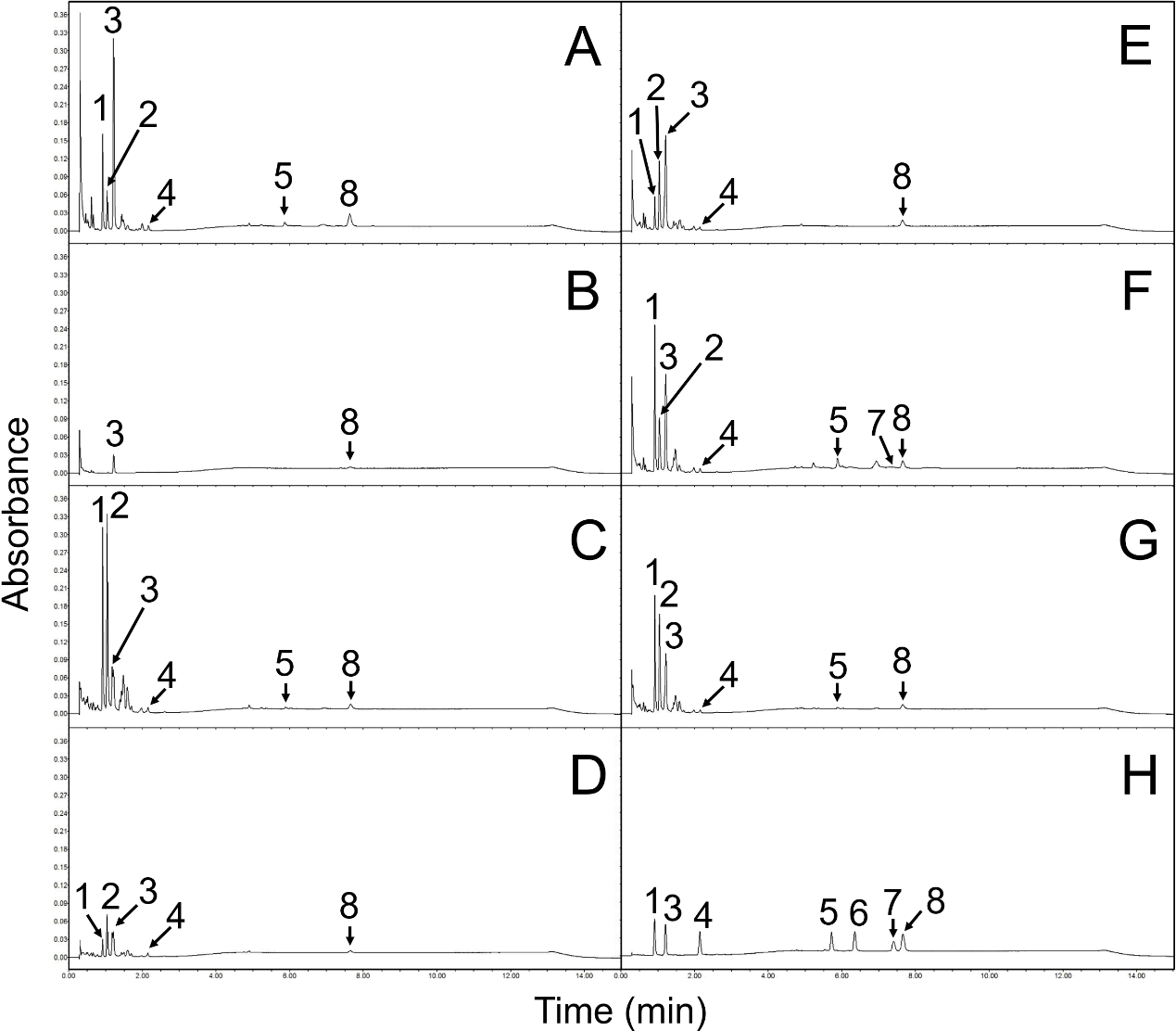

Supplement: Supplementary file 1 [file Image_1.TIF]

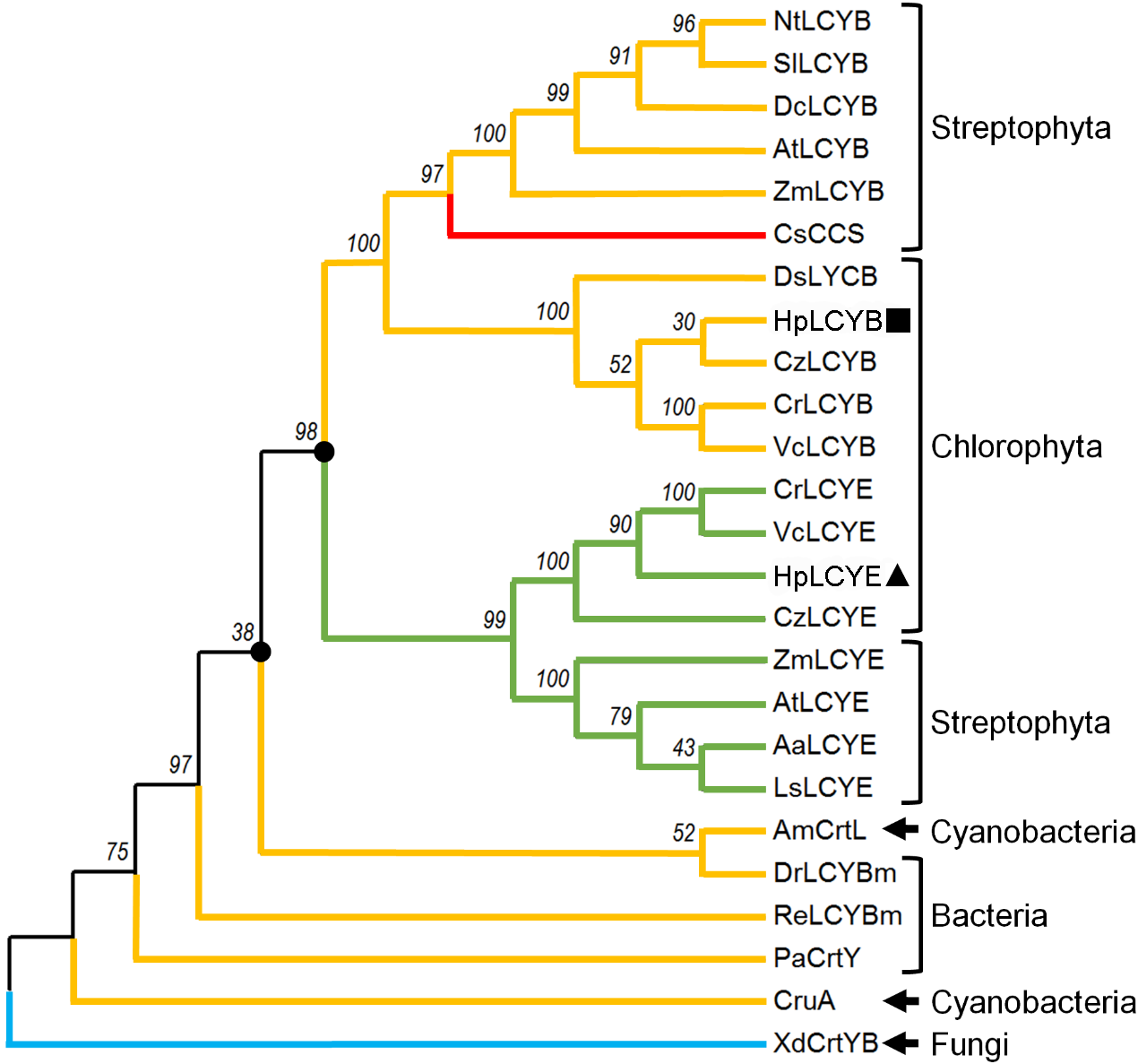

Supplement: Supplementary file 2 [file Image_2.TIF]

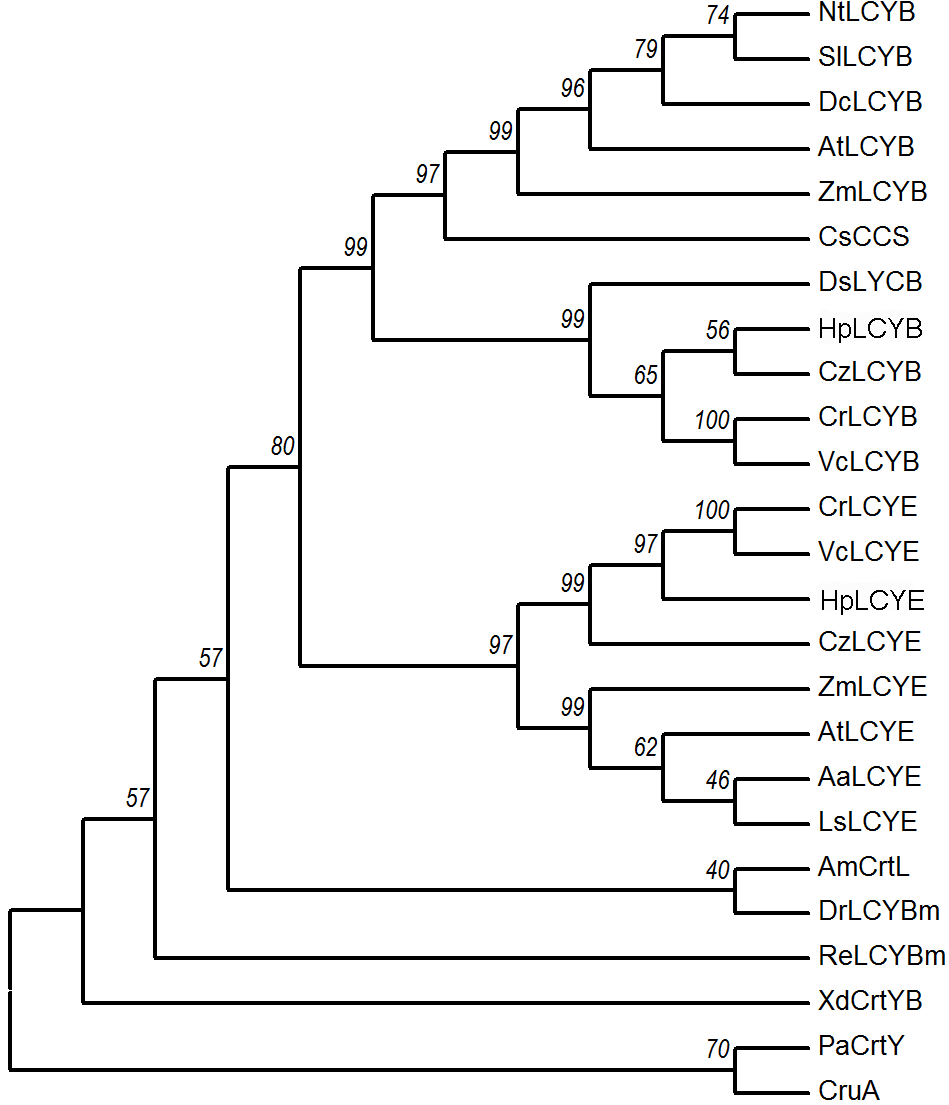

Supplement: Supplementary file 3 [file Image_3.TIF]
