# Supplementary material for: Functional Characterization of a Missing Branch Component in Haematococcus pluvialis for Control of Algal Carotenoid Biosynthesis
Source: Front Plant Sci. 2017 Aug 2;8:1341. doi: 10.3389/fpls.2017.01341 (PMC5539077; doi:10.3389/fpls.2017.01341)
Supplement: Supplementary file 4 [file Table_1.DOCX]

**Supplemental Table S1.** Primers used in this study.

| **Manipulation** | **Primer** | **Sequence (5’ to 3’)** |
| --- | --- | --- |
| **Gene isolation** | | |
| EST isolation | ESTb F | CCMACSTTCTTGTAYGCYATGCC |
|  | ESTb R | MCCWGTTGARGGATGMACCAT |
|  | ESTe F | AACAAYTACGGCGTGTGG |
|  | ESTe R | GGGCCGCCSACMGGGATGTAGCT |
| 3'RACE | 3Rb | CATCAAGGTCAAGGCAGTGGAGG |
|  | 3Re | CTGTTTATGGACTTCAGGCGGCA |
| 5'RACE | 5Rb1 | TCCACTGCCTTGACCTTGATGCC |
|  | 5Rb2 | GAATATCCTGGTCTTGGAGAAGGGC |
|  | 5Re1 | CTGAAGTCCATAAACAGCATCTCGTTG |
|  | 5Re2 | CCTGAAGTCCATAAACAGCATCTCGT |
| cDNA isolation | ORF-B F | ACAGCCCAGATCTG***GGTACC***ATGTTGTCGCCGCTGCAGAG |
|  | ORF-B R | GTGCGGCCGC***AAGCTT***CTACTTGATCATGGCTGGCG |
|  | ORF-E F^a^ | ACAGCCCAGATCTG***GGTACC***ATGGCTGCAGCGCAAGGCAGC |
|  | ORF-E R^a^ | GTGCGGCCGC***AAGCTT***TTAGGCCTGGTTCGGCTCCA |
| **Promoter isolation** | | |
| *LcyB* | LcyB SP1 | CTTTTCAAGCGTGGCCGGTCTACTCG |
|  | LcyB SP2 | TAGGATCGCGCATAACATAGCCTGAC |
| *LcyE* | LcyE SP1 | GCACTAGCTGATTCTGATCCTGAACC |
|  | LcyE SP2 | GACGTTCGGAGTTCACGCAGAATGGCA |
| **Expression plasmid construction** | | |
| pACCRT-EIB-B | EIBYb F | TACCGCATTA***AAGCTT***aggaggattacaaa**ATG**TTGAGCAAACAACACGATT |
|  | EIBYb R | TATCATCGATA***AGCTT***CTACTTGATCATGGCTGGCG |
| pACCRT-EIB-E | EIBYe F | TACCGCATTA***AAGCTT***aggaggattacaaa**ATG**CAACCACTGCGTGTAGCAAC |
|  | EIBYe R | TATCATCGAT***AAGCTT***TTAGGCCTGGTTCGGCTCCA |
| pACCRT-EIBneurE/B | EIBneur F | AAACGCCAGAGTCAGC***CATATG***aggaggattacaaaATGTCCAAGAACACAGAAGG |
|  | EIBneur R | TGTTTCCTGTCGTTTC***CATATG***TCATTGCGCCGCCTTGGC |
|  | Neru F | AAACGCCAGAGTCAGC***CATATG***aggaggattacaaaATGTCCAAGAACACAGAAGG |
|  | Neru R | TGTTTCCTGTCGTTTC***CATATG***TCATTGCGCCGCCTTGGC |
| pACCRT-EIBzetaE/B | EIBzeta F | AAACGCCAGAGTCAGC***CATATG***aggaggattacaaa**ATG**CGCGTAGCGATCGCCGG |
|  | EIBzeta R | TGTTTCCTGTCGTTTC***CATATG***CTATGCCTGACTCGCGGCCA |
|  | Zeta F | AAACGCCAGAGTCAGC***CATATG***aggaggattacaaaATGCGCGTAGCGATCGCCGG |
|  | Zeta R | TGTTTCCTGTCGTTTC***CATATG***CTATGCCTGACTCGCGGCCA |
| pET-32a-LCYE-B | EB F | ACAGCCCAGATCTG***GGTACC***aggaggattacaaa**ATG**CAACCACTGCGTGTAGCAAC |
|  | EBin R | AAATCGTGTTGTTTGCTCAACATtttgtaatcctcctTTAGGCCTGGTTCGGCTCCA |
|  | EBin F | TGGAGCCGAACCAGGCCTAAaggaggattacaaa**ATG**TTGAGCAAACAACACGATTT |
|  | EB R | GTGCGGCCGC***AAGCTT***CTACTTGATCATGGCTGGCGGCA |
| pET-32a-LCYB | LCYB F | ACAGCCCAGATCTG***GGTACC***TTGAGCAAACAACACGATTT |
|  | LCYB R | GTGCGGCCGC***AAGCTT***CTACTTGATCATGGCTGGCGGCA |
| pET-33a-LCYE^a^ | LCYE F | ACAGCCCAGATCTG***GGTACC***CAACCACTGCGTGTAGCAAC |
|  | LCYE R | GTGCGGCCGC***AAGCTT***TCAGGCCTGATTAGGCTCTAGTG |
| **Transcriptional expression study** | | |
| qRT-PCR | qPSY F | CAGCCAGGTGGTCCCTAAACAG |
|  | qPSY R | CTTGGGCGTTATCTTATTGGCG |
|  | qCHYB F | CAACATCATGTGCGATTGGAGG |
|  | qCHYB R | ACAAATAGAATATGGGCGGAGCC |
|  | qLCYB F | TCCAACAACATCCGCATCCAC |
|  | qLCYB R | CCCTAGTTCTTCCCACTCCAGTCAC |
|  | qLCYE F | GCTGCCCTTTGCGGTATTGA |
|  | qLCYE R | TCCTCCTCGTGGACCTTTTTGAC |
|  | qActin F | CACCACTGCTGAGCGGGAGATA |
|  | qActin F | GGAACATTGTAGTCCCGCCTGA |

Sequences overlapping those of vectors are underlined; restriction sites are shown in bold italics; initiation codon (ATG) artificially added is shown in bold; mutant sites are shown in red.

^a^The codons of *HlLcyE* for functional assays have been optimized according to those used in *E. coli*. Here for the full-length cDNA isolation, the original sequence was used for primer design and amplification.
