# Supplementary material for: Functional Characterization of a Missing Branch Component in Haematococcus pluvialis for Control of Algal Carotenoid Biosynthesis
Source: Front Plant Sci. 2017 Aug 2;8:1341. doi: 10.3389/fpls.2017.01341 (PMC5539077; doi:10.3389/fpls.2017.01341)
Supplement: Supplementary file 5 [file Table_2.DOCX]

**Supplemental Table S2.** Sequences used for sequence alignment and molecular evolution analyses.

| **Type** | **Species** | **Accession number** |
| --- | --- | --- |
| Streptophyta LCYB | *Nicotiana tabacum* | NP_001311716 |
|  | *Arabidopsis thaliana* | AAB53337 |
|  | *Zea mays* | AAO18661 |
|  | *Solanum lycopersicum* | ABR57232 |
|  | *Daucus carota* | NP_001316089 |
| Chlorophyta LCYB | *Haematococcus lacustris* | KX424526 (this study) |
|  | *Chlamydomonas reinhardtii* | AAX54906 |
|  | *Chromochloris zofingiensis* | CBH31263 |
|  | *Dunaliella salina* | ACA34344 |
|  | *Volvox carteri f. nagariensis* | EFJ41647 |
| Streptophyta LCYE | *Arabidopsis thaliana* | AAB53336 |
|  | *Adonis aestivalis* | AAK07431 |
|  | *Zea mays* | NP_001146840 |
|  | *Lactuca sativa* | AAK07434 |
| Chlorophyta LCYE | *Chlamydomonas reinhardtii* | XP_001696529 |
|  | *Volvox carteri f. nagariensis* | XP_002945935 |
|  | *Haematococcus lacustris* | AKT95178 (this study) |
|  | *Chromochloris zofingiensis* | CCG06343 |
| Bacterial β monocyclase | Unclassified *Flavobacterium* | BAC77673 |
|  | *Rhodococcus erythropolis* | AAR98749 |
|  | *Deinococcus radiodurans* | AAF10377 |
| Cyanobacterial CrtL | *Acaryochloris marina* | WP_012166204 |
| Eubacterial CrtY | *Pantoea ananatis* | ADD79327 |
| Fungal bi-functional CrtYB | *Xanthophyllomyces dendrorhous* | AAY33923 |
| Cyanobacterial CruA | *Synechococcus* sp. | WP_012307753 |
| Streptophyta CCS | *Citrus sinensis* | AAF18389 |

CCS: capsanthin-capsorubin synthase
